# Effects of Dietary Supplementation with Glutamate and Aspartate on Diquat-Induced Oxidative Stress in Piglets

**DOI:** 10.1371/journal.pone.0122893

**Published:** 2015-04-15

**Authors:** Jie Yin, Mingfeng Liu, Wenkai Ren, Jielin Duan, Guan Yang, Yurong Zhao, Rejun Fang, Lixiang Chen, Tiejun Li, Yulong Yin

**Affiliations:** 1 Scientific Observing and Experimental Station of Animal Nutrition and Feed Science in South-Central, Ministry of Agriculture, Hunan Provincial Engineering Research Center of Healthy Livestock, Key Laboratory of Agro-ecological Processes in Subtropical Region, Institute of Subtropical Agriculture, Chinese Academy of Sciences, Changsha, Hunan, 410125, China; 2 University of Chinese Academy of Sciences, Beijing, 100039, China; 3 Department of Animal Science, University of Hunan agriculture, Changsha, 410128, China; 4 Department of Animal Science, University of Florida, Gainesville, Florida, 32610, United States of America; 5 Southwest Collaborative Innovation Center of Swine for Quality & Safety, 211#211 Huiming Road, Wenjiang district, Chengdu, China; The Ohio State Unversity, UNITED STATES

## Abstract

This study aimed to investigate the protective effects of dietary glutamate and aspartate supplementations on diquat-induced oxidative stress in piglets. Diquat injection significantly reduced growth performance, including body weight, average daily weight gain, and feed intake (P<0.05). Meanwhile, diquat administration induced oxidative stress evidenced by the decreased serum nitric oxide (NO) and elevated malondialdeyhde (MDA) concentration (P<0.05). Furthermore, diquat-induced oxidative stress disrupted intestinal absorption system and decreased serum threonine, serine, and glycine levels. Dietary supplementation with glutamate improved final body weight, antioxidant system, and expressions of amino acids transporters and enhanced serum glutamate concentration compared with diquat group (P<0.05). While aspartate failed to alleviate diquat-induced oxidative stress, growth depression, and dysfunction of nutrients absorption except for liver relative weight. In conclusion, dietary supplementation with glutamate confers beneficial effects on diquat-induced oxidative stress in piglets, while aspartate exhibits little effects.

## Introduction

Oxidative stress can be induced by various factors during the animal growth and development, including physical (weaning, housing, transport, and novel handling), social (relocation with unfamiliar penmates), and pathological environments [1–3]. We found that pathological factors such as mold-contaminated feed, porcine circovirus type 2 infection, and dextran sulfate sodium-induced colitis exhibit an inhibitory effect on activities of antioxidant enzymes and induce oxidative stress in pigs and mice [3–5]. Our latest studies also revealed that birth and weaning processes disrupt oxidative balance and cause oxidative injury in piglets [2,6]. Oxidative stress correlates with the modification of protein, lipid oxidation, and nucleic acid breaks, and compelling evidences have demonstrated that oxidative stress involves in the development of many diseases [1].

Glutamate and aspartate are functional amino acids and have been shown to exert various functions in nutrients metabolisms, energy requirements, immune responses, oxidative stress, regulation of signaling pathways, and synaptic transmitting [7–9]. Dietary supplementation with glutamate exhibits a beneficial role in deoxynivalenol and mycotoxins challenged pigs [10,11]. Furthermore, Pi et al. reported that dietary supplementation of aspartate enhances intestinal integrity and energy status in weanling piglets after lipopolysaccharide challenge [12]. However, little is known about effects of glutamate and aspartate on oxidative stress. Diquat has been widely used to induce oxidative stress *in vivo* and injection of diquat exerts inhibitory effects on growth performance [13] and nutrients metabolism [14]. Thus, the current study was to investigate the protective roles of dietary glutamate and aspartate in diquat-induced oxidative stress in piglets.

## Materials and Methods

This study was conducted according to the guidelines of the Declaration of Helsinki and all procedures involving animal subjects were approved by the animal welfare committee of the Institute of Subtropical Agriculture, University of Chinese Academy of Sciences [13].

Twenty-four healthy piglets of similar body weight (9.92 ± 0.30 kg) (Landrace× Large White) (ZhengHong Co., China) were randomly divided into four groups (n = 6): one control group (control), one diquat group (diquat), one glutamate group in which piglets were fed 2% glutamate (glutamate group), and one aspartate group in which piglets received 2% aspartate (aspartate group). All piglets were fed basal diet for 5 days, then injected intraperitoneally (i.p.) with either 10 mL saline or 10 mg/kg body weight diquat in 10 ml saline to induce oxidative stress according to previous report [14]. After injection of diquat, feed in glutamate and aspartate groups was added 2% glutamate and 2% aspartate, respectively. Feed intake was recorded daily to calculate average daily feed intake (ADFI). After 7 days of experimental period, body weight was weighed and blood was sampled from a jugular vein before slaughter [15]. All piglets were anesthetized with sodium pentobarbital and killed by jugular puncture [16]. The basal diet was prepared from corn, soybean meal, wheat bran, limestone, CaHPO_4_, NaCl, and additive premix to meet or exceed the nutritional requirements of piglets according to our previous report [17]. Glutamate and aspartate were added to the feed and mixed uniformly.

### Calculation of relative organ weights

The heart, liver, spleen, and kidney were separated and weighed. The relative organ weights were calculated basing the ratio of organ weigh to body weight [18].

### Measurements of oxidative stress index

Blood samples were centrifuged at 3000 × g for 10 min and 4°C, and supernatant were collected for serum analysis [19]. Superoxide dismutase (SOD), glutathione peroxidase (GSH-Px), nitric oxide (NO), and malondialdeyhde (MDA) in serum were measured using spectrophotometric kits in accordance with the manufacturer’s instructions (Nanjing Jiangcheng Biotechnology Institute, China) [20].

### Histomorphometry determination

Samples from jejunal and ileal middle section (3cm) were kept in 4% neutral buffered 10% formalin for H&E staining. Villus height and crypt depth were measured using an image-analysis system [4].

### Determination of serum amino acids

Amino acids in serum were determined by LC–MS/MS (HPLC Ultimate3000 and 3200 QTRAP LC–MS/MS) according to our previous report [4].

### Real time PCR (RT-PCR)

Extraction of total RNA and its reverse transcription were performed according to our previous reports [4,6]. Primers were designed with Primer 5.0 according to the gene sequence of pig (http://www.ncbi.nlm.nih.gov/pubmed/) to produce an amplification product (Table 1). β-actin was used as a housekeeping gene to normalize target gene transcript levels. Real-time PCR was performed according to our previous studies [4,6]. Relative expression was normalized and expressed as a ratio to the expression in control group.

**Table 1:** Primers used in this study.

Gene	Accession No.	Primer squence (5’-3’)	Size (bp)
SLC1A1	NM_001164649.1	F:GGCACCGCACTCTACGAAGCA	177
		R:GCCCACGGCACTTAGCACGA	
SLC7A1	NM_001012613.1	F: TGCCCATACTTCCCGTCC	192
		R:GGTCCAGGTTACCGTCAG	
NAAT	XM_003355984.2	F:GATTGTGGAGATGGAGGATGTG	128
		R:TGCGAGTGAAGAGGAAGTAGAT	
β-actin	XM_003124280.3	F:CTGCGGCATCCACGAAACT	147
		R:AGGGCCGTGATCTCCTTCTG	

F: forward primer; R: reverse primer; SLC1A1: solute carrier family 1, member 1; SLC7A1: solute carrier family 7, member 1; NAAT: neutral amino acid transporter.

### Statistical Analysis

All data were analyzed using Grubbs’ test and then performed by using the one-way analysis of variance (ANOVA) to test homogeneity of variances via Levene’s test and followed with Ducan’s multiple comparison test (SPSS 17.0 software). Data are expressed as the mean ± standard error of the mean. Values in the same row with different superscripts are significant (P < 0.05), while values with same superscripts are not significant different (P > 0.05) [21].

## Results

### Effects of glutamate and aspartate on growth performance in diquat-challenged piglets

The results of growth performance were summarized in Table 2 and S1 Dataset. Injection of diquat significantly decreased final body weight, average daily weight gain, and average daily intake (P < 0.05). Meanwhile, supplementation with glutamate restored the inhibitory effect of body weight caused by diquat (P < 0.05).

**Table 2:** Growth performance and relative organ weights in four groups.

Item	Control	Diquat	Glutamate	Aspartate	SE	p-value
Initial body weight (kg)	9.88±0.70	9.90±0.61	9.98±0.65	9.91±0.59	0.30	0.999
Final body weight (kg)	11.36±0.62^a^	8.87±0.75^b^	10.31±0.50^a^	8.82±0.47^b^	0.44	0.009
Average daily weight gain (kg)	0.32±0.05^a^	-0.08±0.04^b^	-0.06±0.01^b^	-0.12±0.04^b^	0.05	<0.001
Average daily intake (kg)	0.61±0.07^a^	0.26±0.07^b^	0.32±0.03^b^	0.26±0.05^b^	0.04	0.001
Heart (%)	4.68±0.09	6.17±0.66	5.57±0.27	6.37±0.78	0.30	0.196
Liver (%)	26.31±0.29^b^	30.80±0.84^a^	31.72±1.93^a^	26.27±1.66^b^	0.84	0.017
Spleen (%)	2.02±0.05	1.90±0.12	2.05±0.09	2.03±0.07	0.04	0.599
Kidney (%)	5.20±0.22	6.44±0.58	6.28±0.51	6.10±0.37	0.23	0.225

^a,b^Within a row, means with different superscripts differ (P<0.05). The same as below.

**Fig 1:**
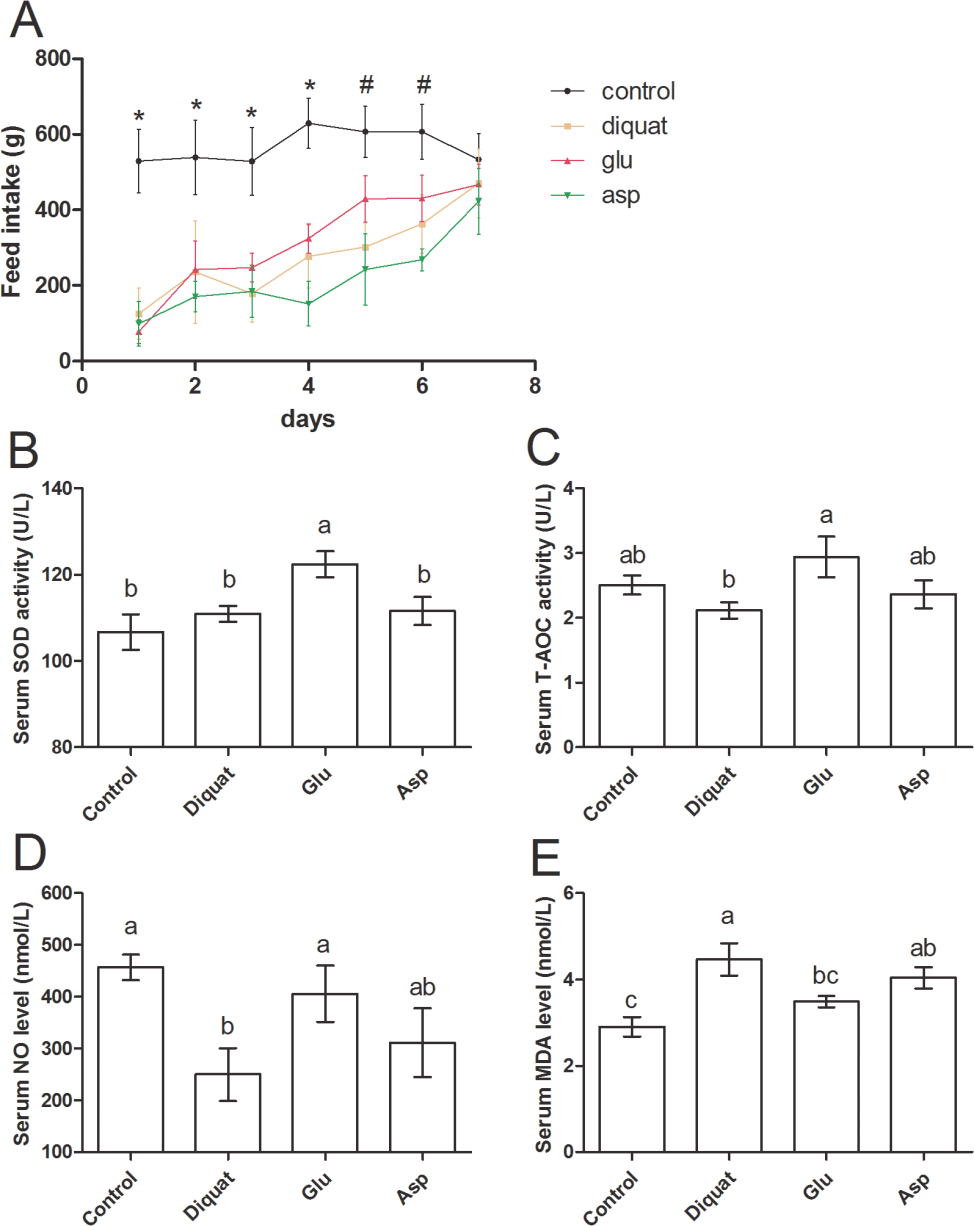
Feed intake (A) and serum SOD, T-AOC, NO, and MDA levels (B, C, D, and E) in four groups after exposure to diquat. * means feed intake in control is significantly higher than that in other three groups and # means feed intake in control is markedly higher than that in diquat and aspartate groups (P<0.05), but has no difference compared with glutamate group (P>0.05).

In addition, we monitored the feed intake in all piglets after challenging diquat (Fig 1 A and S1 Dataset). Feed intake was markedly lower after diquat injection compared with control group at day 1 to day 6 (P < 0.05). While at day 5 and 6, feed intake in glutamate group was much higher than that in diquat group (P > 0.05). At day 7, all piglets challenged diquat recovered to normal intake (P > 0.05).

### Effects of glutamate and aspartate on relative organ weights in diquat-challenged piglets

The effects of glutamate and aspartate on relative organ weights in diquat-challenged piglets were shown in Table 2 and S1 Dataset. There was no difference in heart, spleen, and kidney relative weights between four groups (P > 0.05), while diquat injection significantly increased liver relative weight (P < 0.05). Although supplementation with glutamate failed to exhibit a protective effect on alteration in liver relative weight caused by diquat (P > 0.05), aspartate significantly reduced the liver relative weight compared with diquat group (P < 0.05).

### Effects of glutamate and aspartate on oxidative stress in diquat-challenged piglets

Diquat has been reported to induce oxidative stress in mice [22] and pigs [14], so we next determined serum oxidative stress-related indexes, including SOD, T-AOC, NO, and MDA (Fig 1 and S2 Dataset). The results showed that serum SOD activity was not changed after exposure to diquat, while supplementation with glutamate significantly increased SOD activity compared with other groups (P < 0.05). Diquat administration tended to block serum T-AOC (P > 0.05) and glutamate markedly restored (P < 0.05) the inhibitory function compared with diquat qroup. NO, a gas signal related to oxidative stress [23], markedly decreased after exposure to diquat (P < 0.05), dietary supplementation with glutamate increased serum NO level compared with diquat group (P < 0.05). MDA is a metabolite of lipid oxidation [24] and was significant higher in diquat group than that in control piglets. Similarly, glutamate supplementation significantly reduced the MDA level compared with diquat qroup (P < 0.05). However, in the present study, we failed to notice any significant difference in serum SOD, T-AOC, NO, and MDA levels after dietary supplementation with aspartate (P < 0.05).

### Effects of glutamate and aspartate on intestinal morphological structure in diquat-challenged piglets

**Fig 2:**
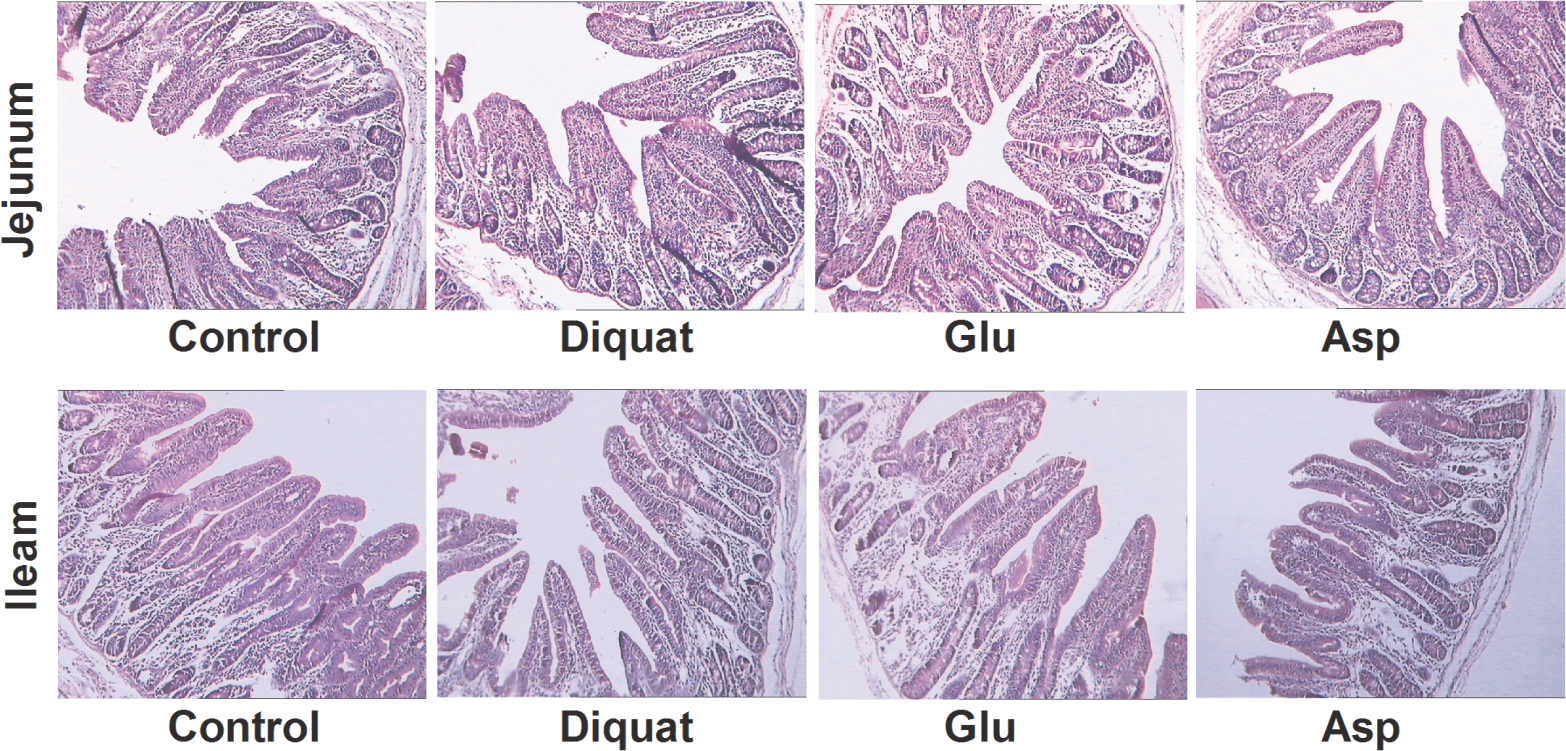
Histological evaluation of intestinal tissues (HE×250) after exposure to diquat.

The HE staining results shown at Fig 2, we found that diquat injection failed to cause intestinal morphological injury. We further calculated the intestinal villus height and crypt depth in four groups (Table 3 and S3 Dataset) and there was no difference (P > 0.05).

**Table 3:** The intestinal villus height and crypt depth in four groups.

Item(um)	Control	Diquat	Glutamate	Aspartate	SE	p-value
Jejunam						
villus height	385.82±21.19	402.55±27.18	357.94±10.32	375.56±15.60	10.23	0.507
Crypt depth	179.00±13.75	175.18±10.55	184.03±15.76	173.20±8.08	5.92	0.930
V/C	2.19±0.13	2.16±0.13	2.13±0.18	2.044±0.12	0.07	0.899
Ileam						
Villus height	389.33±27.63	391.63±23.79	370.95±31.49	337.28±12.06	12.43	0.404
Crypt depth	181.20±22.18	185.75±21.56	142.56±20.57	147.53±10.57	9.84	0.294
V/C	2.24±0.18	2.03±0.18	2.24±0.20	2.21±0.08	0.08	0.806

### Effects of glutamate and aspartate on serum free amino acids in diquat-challenged piglets

Serum free amino acids analysis after diquat exposure was shown at Table 4 and S3 Dataset. Compared with control group, threonine, serine, and glycine concentrations were significant lower in diquat-challenged piglets (P < 0.05). Although supplementation with glutamate failed to restore the threonine, serine, and glycine concentrations, it significantly enhanced serum glutamate level (P < 0.05). However, aspartate failed to affect serum amino acids concentration (P > 0.05).

**Table 4:** Serum free amino acids in four groups.

Item	Control	Diquat	Glutamate	Aspartate	SE	p-valve
Aspartate	134.14±12.03	111.65±16.01	149.42±15.10	115.59±13.33	7.34	0.776
Threonine	842.33±31.23^a^	490.00±95.47^b^	658.88±58.44^ab^	473.99±90.93^b^	46.39	0.096
Serine	265.65±13.90^a^	188.56±17.94^bc^	227.00±20.31^ab^	174.59±13.29^c^	10.71	0.863
Glutamate	721.79±45.89^ab^	681.63±43.59^b^	872.23±74.57^a^	707.58±38.32^b^	28.99	0.118
Glycine	1548.84±74.86^a^	1143.86±148.21^b^	1286.86±162.39^ab^	960.69±85.48^b^	73.05	0.410
Alanine	912.77±30.54	937.18±92.15	1159.52±93.53	874.15±144.37	51.55	0.115
Cysteine	35.35±2.80^b^	47.34±3.87^ab^	57.55±4.18^a^	49.26±6.41^ab^	2.68	0.062
Valine	187.19±23.30	214.78±16.33	225.52±28.11	236.80±29.15	12.16	0.590
Methionine	75.57±9.37	51.19±7.89	75.89±11.61	50.78±2.29	4.74	0.389
Isoleucine	100.83±8.79	113.14±8.76	112.55±7.74	113.66±11.97	4.54	0.659
Leucine	214.20±13.01	244.83±10.19	261.60±24.51	231.67±15.07	8.54	0.140
Tyrosine	64.49±9.90	47.92±5.18	58.77±5.91	44.25±4.58	3.56	0.591
Phenylalanine	137.52±6.14	133.79±9.75	138.19±6.09	131.80±7.79	3.58	0.666
Lysine	472.97±56.37	372.02±49.98	399.15±55.32	372.37±28.24	24.33	0.499
NH3	780.68±25.52	804.27±29.82	861.79±35.81	785.66±35.18	16.30	0.793
Histidine	92.60±12.51	83.18±6.54	109.84±11.89	101.12±8.86	5.20	0.357
Arginine	189.58±17.31	195.50±15.41	203.23±19.81	188.10±27.67	11.37	0.402
Proline	359.98±19.23	332.53±40.63	409.30±37.12	306.43±33.24	17.54	0.721

### Effects of glutamate and aspartate on intestinal relative gene expressions in diquat-challenged piglets

**Fig 3:**
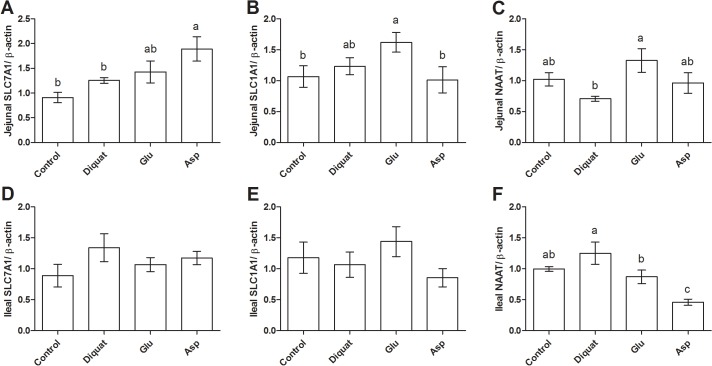
Intestinal relative mRNA abundances in four groups after exposure to diquat.

We next preformed RT-PCR to test expressions of solute carrier family 7, member 1 (SLC7A1), solute carrier family 1, member 1 (SLC1A1), and neutral amino acid transporter (NAAT) in the intestine in all piglets (Fig 3 and S4 Dataset). The results exhibited that diquat injection had no effects on these transporters expression in the intestine (P > 0.05). While supplementation with glutamate markedly up-regulated jejunal SLC1A1 expression compared with control group and enhanced jejunal NAAT mRNA abundance compared with diquat group (P < 0.05). Glutamate addition significantly down-regulated ileal NAAT expression compared with diquat treatment (P < 0.05). Furthermore, dietary supplementation with aspartate significantly enhanced jejunal SLC7A1 mRNA abundance compared with the control and diquat group (P < 0.05), while inhibited ileal NAAT expression compared with other groups (P < 0.05).

## Discussion

Diquat, a bipyridyl herbicide, can convert molecular oxygen into superoxide anion radical and stimulate cellular production of free radical species via undergoing cyclic reduction–oxidation processes. Thus, diquat has been widely used to induce animal oxidative stress *in vivo* and diquat-induced oxidative stress has been reported to affect growth performance and nutritional metabolism [14,25,26]. For example, Lv *et al*. found that 10 mg/kg diquat significantly enhances serum MDA concentration and inhibited activity of antioxidant enzymes, including SOD and GSH-Px [14]. In the present study, administration of diquat markedly decreased serum NO level and increased serum MDA concentration, indicating a significant disruption in the oxidative balance after exposure to diquat injection.

Glutamate has been demonstrated to improve growth performance and health in pigs [27–32], and we found that supplementation with glutamate significantly increased the final body weight compared with diquat group, suggesting a potentially important role for glutamate in mitigating adverse effects of oxidative stress on piglets. Furthermore, Pi et al. reported that addition of aspartate alleviates growth suppression of weaned pigs after the LPS challenge [12], while the present study failed to exhibit significant difference in growth performance after dietary aspartate. The reason might be different model and different dosage of aspartate.

Our previous reports have shown that supplementation with glutamate can restore deoxynivalenol and mycotoxins induced oxidative stress in pigs [10,11]. The present data indicated that glutamate alleviated diquat-induced oxidative stress via enhancing SOD, T-AOC, and NO levels and inhibiting lipid oxidation subsequent with MDA generation. It is widely recognized that glutamate exhibits an antioxidant function as glutamate is a precursor for glutathione (GSH) along with cysteine and glycine [33]. In our lab, we have found that glutamate is the main limiting substrate compared with cysteine and glycine for liver GSH synthesis in mice (unpublished data). GSH homeostasis in body is an important cellular defense against oxidative stress and involves in the cellular redox state and in the detoxification process [1]. Thus, we speculated glutamate alleviates diquat-induced oxidative stress in piglets through increasing GSH synthesis mechanism. But further data about the serum and hepatic GSH synthesis function are needed to validate this explanation.

Diquat-induced oxidative stress decreased serum threonine, serine, and glycine concentrations in the present study. It is likely that degradation and metabolism of dietary threonine, serine, and glycine in the intestine are increased in response to the oxidative stress, resulting in their deficiencies in piglets. Various reports have shown that the serine/threonine kinase and these amino acids metabolisms are essential for oxidative stress response [34–35] and glycine can reduce the oxidative stress and elevate the enzymic and non-enzymic antioxidants in animals [36]. The current study also showed that supplementation with glutamate increased serum glutamate concentration after diquat challenge. An increase in the influx of glutamate from the lumen of the small intestine into the enterocyte can enhance tissue protein synthesis and improve antioxidant system via enhancing GSH synthesis.

The amino acids transporters mainly contribute to the absorption of luminal amino acids into serum [37]. Thus, we next measured several transporters expressions in the intestine after exposure to diquat-induced oxidative stress. Although there were no differences in mRNA abundances for SLC7A1, SLC1A1, and NAAT between control and diquat groups, supplementation with glutamate and aspartate affected these transporters expression in the intestine, which may mediate glutamate absorption in response to diquat-induced oxidative stress. Indeed, our previous reports have indicated that dietary supplementation with amino acids modulate intestinal activities of nutrients transporters in pathological conditions [4,10, 38].

## Supporting Information

S1 Dataset Growth performance and relative organ weight in four groups after exposure to diquat. (XLSX)

S2 Dataset Oxidative stress indexes in four groups after exposure to diquat. (XLSX)

S3 Dataset Intestinal morphological structure and serum amino acids profiles in four groups after exposure to diquat. (XLSX)

S4 Dataset Expression of intestinal amino acid transporters in four groups after exposure to diquat. (XLSX)
